# Effect of Socioeconomic Strata and Land Cover on Dengue Hotspots in Medellin, Colombia

**DOI:** 10.4269/ajtmh.24-0665

**Published:** 2025-03-18

**Authors:** Juliana Pérez-Pérez, John Alexander Pulgarin Diaz, Guillermo Rúa-Uribe, Blas Mola-Yudego, Eric Delmelle, Raúl Rojo, Frank Berninger

**Affiliations:** ^1^ Department of Environmental and Biological Sciences, Joensuu Campus, University of Eastern Finland, Joensuu, Finland;; ^2^ School of Forest Sciences, University of Eastern Finland, Joensuu, Finland;; ^3^ Corporación Colombiana de Investigación Agropecuaria — AGROSAVIA, Centro de Investigación El Nus — Vereda ICA, Corregimiento San José del Nus, municipio de San Roque, Antioquia, Colombia;; ^4^ Grupo Entomología Médica, Facultad de Medicina, Universidad de Antioquia, Medellín, Colombia;; ^5^ Biostatistics and Health Data Science, Lehigh University, Bethlehem, Pennsylvania;; ^6^ Cartography and Geographic Information Systems Group, Vrije Universiteit Brussels, Brussels, Belgium;; ^7^ Urban Collaborative Health, Dornsife School of Public Health, Drexel University, Philadelphia, Pennsylvania;; ^8^ Programa de Control de Vectores, Secretaría de Salud, Alcaldía de Medellín, Medellín, Colombia

## Abstract

Despite extensive vector control programs, dengue remains a significant global health challenge, with outbreaks rising worldwide. Effective dengue control requires reinforcing the surveillance systems and using surveillance data to gain a better understanding of dengue dynamics at both spatial and temporal scales. We studied the effect of socioeconomic and land cover on the presence of dengue hotspots in Medellin (Colombia) from 2010 to 2020 and identified recurrent hotspots during severe epidemic (SE), epidemic (E), and non-epidemic (NE) years. We focused on spatial autocorrelation using global and local indicators of spatial association over 40,814 georeferenced dengue cases. Later, we tested if the spatial units identified as hotspots, recurrent hotspots, and non-hotspots were evenly distributed among socioeconomic strata and land cover categories. During the study period, 50% of the dengue cases were concentrated in 26% of the study area. We identified statistically significant hotspots, some recurring for up to 7 years with their spatial patterns differing between SE, E, and NE years, even though some recurred over time. Recurrent hotspots predominantly occurred in low–medium socioeconomic strata and were absent in the highest strata. Also, they predominated in human-made structures. The interaction between socioeconomic factors, land cover, and potentially, the vector presence seems to explain the spatial variation of dengue epidemics and their recurrent hotspots in Medellin.

## INTRODUCTION

Mosquito-borne diseases represent a significant global health threat, with dengue as a concerning example of a rapidly spreading disease worldwide. Dengue virus has four serotypes and is transmitted to humans through the bites of infected female mosquitoes *Aedes* (*Stegomyia*) *aegypti* (L.) and *Aedes* (*Stegomyia*) *albopictus* (Skuse) (dengue vectors)*.* Dengue is present in more than 100 countries with 390 million individuals infected per year, with half of the world’s population at risk.^1^ According to WHO, from 2000 to 2019, there has been a 10-fold increase in reported cases globally, increasing from 500,000 to 5.2 million. Climate change, deforestation, and urbanization are some of the major risk factors behind the increasing number of outbreaks (a sudden increase in the number of cases) of dengue and other mosquito-borne diseases.^2^ As a result, dengue is now endemic in many countries across Africa, the Americas, the eastern Mediterranean, Southeast Asia, and the western Pacific.^2^ The increasing occurrence of dengue in these regions as well as its recent emergence in previously transmission-free areas in Europe highlights its significant public health concern.^3^

In Latin American cities, the burden of dengue is pronounced, with several areas experiencing recurrent outbreaks and high rates of transmission.^4^ The environment and socioeconomic conditions in urban areas are heterogeneous, with fast and unplanned urbanization favoring the spread of mosquito-borne diseases.^5^ In addition, in some areas, outbreaks are further influenced by the cocirculation of both vectors alongside the simultaneous circulation of all four serotypes of the dengue virus.^6^

Because of the lack of dengue-specific treatments and the limited availability of globally licensed vaccines (with only two vaccines approved for specific age groups in a few countries), emphasis has been given to surveillance and control programs based on an integrated management strategy, which includes entomological, viral, serological, and clinical surveillance.^7^ However, limited resources lead to control efforts focused on reducing the abundance of mosquitoes and breeding sites and managing local outbreaks using chemical control.^8^ During major dengue outbreaks, these programs display a reactive response targeting the surrounding outbreak areas, which has failed because the surveillance system is prone to delayed case reporting and underreporting.^9^

In endemic cities, cases concentrate in certain areas or “hotspots,” highlighting the spatial heterogeneity of transmission,^10^ where a *spatial risk stratification* approach could prioritize areas to implement a preventive strategy.^8^^,^^11^ This approach assumes that preventive measures in hotspots could reduce the risk of exposure and transmission, containing epidemics and lowering incidence. Hotspot-targeted interventions to control malaria have shown mixed evidence of effectiveness, with some studies reporting positive outcomes and others highlighting methodological limitations that affect the interpretation of intervention impact.^12^ Unfortunately, these interventions have not been documented for dengue.

To prioritize areas where to implement preventive strategies and control dengue transmission, the first step is to identify the main triggers of significant hotspots for informed decision-making in controlling the burden of dengue in Medellin. For this, 1) we identified statistically significant dengue hotspots in the urban area of Medellin (Colombia); 2) examined their recurrence across severe epidemic (SE), epidemic (E), and non-epidemic (NE) years; 3) evaluated the hotspot distribution based on the socioeconomic strata and land cover variables; and 4) set the basis to recommend strategies for prioritizing control interventions in hotspots areas, contributing to a more efficient allocation of resources for dengue control in hyperendemic cities.

## MATERIALS AND METHODS

### Study area.

Medellin, the second most populated city in Colombia (South America) with approximately 2.5 million inhabitants, is located at an altitude of approximately 1,479 meters above sea level. It has a tropical climate characterized by an average temperature of 24°C (with an average minimum of 15°C and an average maximum of 30°C). Its annual rainfall ranges from 1,500 to 2,500 mm.^13^

**Figure 1.**
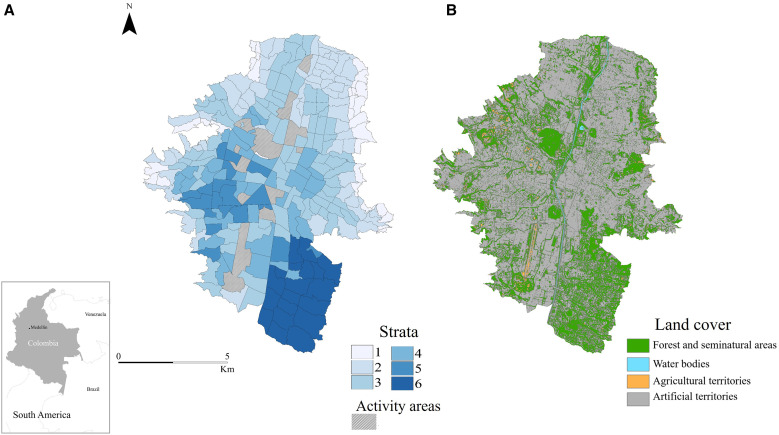
Study area: Medellin (Colombia). (**A**) Classification of the spatial units by socioeconomic strata from one (lowest) to six (highest). (**B**) Land cover use.

The city is divided into 249 neighborhoods (*barrio* in Spanish; hereafter referred to as *spatial unit*), which are classified into six levels (strata) according to the Colombian Government’s scale used for socioeconomic classification (Figure 1A). This classification serves as a useful indicator of the urban population’s socioeconomic status in Colombia.^14^^,^^15^ It is also used for taxation on public services, like water, sewer, electricity, and gas.^16^ The lowest strata (strata 1 and 2) are found in the northern part of the city, whereas the middle strata (strata 3 and 4) predominate in the center, and the highest strata (strata 5 and 6) concentrate in the southeast.^14^ In the lower strata, the availability of green spaces has decreased more in recent years compared with medium- and high-socioeconomic strata, possibly because of urban densification in those areas (Figure 1B).

### Data sources.

We used dengue symptomatic records from 2010 to 2020 recorded through the National Public Health Surveillance System of Colombia. We used a deidentified dataset provided by the Local Surveillance Department in the Secretariat of Health; therefore, informed consent was not required as only secondary data were used.

The geocoding of dengue cases in Medellin is challenging because the address information was registered in a variety of formats, such as in other cities in Colombia.^17^^,^^18^ To solve this, we depurated the database using an automatic geocoding strategy and our knowledge of the city. For this, we used the geocoding service provided through the Map Service of the Municipality of Medellin^19^ and manual geocoding strategies, assuring geocoding precision at the house level. Initially, 52,009 cases were processed, from which 8,628 were not geocoded and 2,657 cases were located outside the study area (Figure 1), resulting in a final dataset of 40,814 geocoded cases within the urban area of Medellin for spatial analysis.

To assess socioeconomic and land cover variables, we used the established classification systems: the Colombian Government’s scale for socioeconomic status (Figure 1A)^20^ and the Coordination of Information on the Environment-based land cover classification adapted for Colombia (Figure 1B).^21^ Colombia adapted its land cover classification with a hierarchical structure with four categories: 1) artificial surfaces, which include areas primarily occupied by human-made structures, such as urban fabric, industrial areas, and transport infrastructure; 2) agricultural areas, which include areas for farming (e.g., arable land, permanent crops, and pastures); 3) forests and seminatural areas, which include forested regions, woodlands, and other natural habitats that may have been influenced by human activity; and 4) water bodies, including lakes, rivers, and other bodies of water both natural and artificial. We calculated the proportion of land cover categories in each spatial unit to compare between hotspot classes. All of these variables were obtained from the Open Data Portal of the Medellin Municipality.

## STATISTICAL ANALYSES

Aiming to identify hotspots of different virus transmission, some studies use incidence rates (number of cases per at-risk population) instead of case counts because the latter is influenced by the at-risk population when the risk is constant.^22^ For example, a high at-risk population may lead to a higher number of cases, and a low at-risk population may lead to a lower number of cases. However, using incidence rates has its challenges; for example, in areas with low at-risk populations, even a few cases can inflate the rates, whereas in high-population areas, the same number of cases deflates the rates.^23^^,^^24^ Some researchers propose using empirical Bayes methods to solve this instability,^25^ but this approach can reduce the impact of high case numbers and increase the impact of low case numbers when identifying hotspots, potentially concealing them.^24^ Given these, some epidemiological studies used the number of cases to identify hotspots, assessing the risk that each case represents for the disease spread.^10^^,^^26^^–^^28^ In addition, Colombia and other Latin American countries account for the number of cases as the immediate concern lies in avoiding outbreak scenarios,^26^^–^^28^ where a fast response is needed.

**Figure 2.**
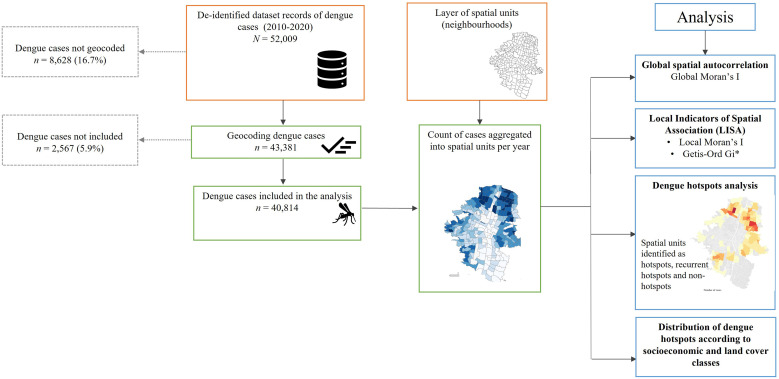
Methodological flowchart for assessing spatiotemporal dynamics of dengue hotspots in Medellin (Colombia) from 2010 to 2020.

Using previous research detecting dengue hotspots^10^^,^^26^^–^^28^ and the technical guidance to implement a dengue risk stratification approach,^8^ we aggregated the 40,814 geocoded cases into 249 spatial units. Although it is still an artificially imposed setting, it is practical because dengue surveillance and prevention programs are often conducted at this level for the studied city and others in Colombia.^17^^,^^18^^,^^29^ A summary of the applied methods is depicted in Figure 2.

### Spatial autocorrelation.

Spatial autocorrelation (SA) quantifies the degree to which a variable is correlated with itself across space.^30^ To examine it, we used global Moran’s index (*I*)^31^ using the aggregated dengue cases and investigate whether these units are random, dispersed, or aggregated. This widely used index evaluates if neighboring units tend to exhibit similar values. However, it is global and does not indicate where autocorrelation occurs.^31^ It is centered on the mean, meaning that the model has a constant mean, and areal patterns are caused by the possible spatial relationship between areal units, indicated by the spatial weights or the importance given to their relationship.^23^^,^^32^ Moran’s *I* ranges from −1 to 1, where −1 indicates strong negative SA, 0 indicates complete spatial randomness, and 1 strong positive SA.^30^^,^^33^

We computed global Moran’s *I* yearly to account for the variability in the number of cases across the studied years, which were classified as NE, E, and SE based on endemic channels of dengue from health authorities in Colombia (Table 1).

**Table 1:** Classification of the studied years according to the number of dengue cases in Medellin (Colombia) from 2010 to 2020

Epidemic Level (abbreviation)	Number of Cases	Years of Analysis
Non-epidemic (NE)	0–1,999	2011, 2012, 2018, 2019, and 2020
Epidemic (E)	2,000–9,999	2013, 2014, 2015, and 2017
Severe epidemic (SE)	10,000–18,000	2010 and 2016

We used a fixed distance band of 1 km to select spatial units for comparison, ensuring that all spatial units would have at least one neighbor (even between fragmented residential areas). We set the dependency degree between spatial units (spatial weights) using a binary strategy, with the value of one if the spatial unit is within the distance; otherwise, the value was zero. After obtaining the neighbors and their weights, we calculated Moran’s *I* using 9,999 permutations (α = 0.05).^33^

### Local indicators of spatial association.

To identify dengue hotspots, we used local indicators of spatial association (LISAs). Unlike global Moran’s *I*, which summarizes the overall SA over the study area in a single index, LISA methods study the SA at the local level.^30^ There are different LISA methods, from which we selected local Moran’s *I* and Getis Ord-Gi*. The main difference between these two methods is that the local mean for Moran’s *I* includes only neighboring spatial units, whereas the local mean for Getis-Ord Gi* includes neighboring spatial units and the one in question.^33^^–^^35^ In addition, local Moran’s *I* needs the use of permutations to generate a reference distribution to assess the statistical significance of observed patterns, whereas Getis-Ord Gi* assumes normality and provides a direct measure of significance.^33^^–^^35^ By using both methods, we can achieve a more comprehensive understanding of the hotspots distribution.

Local Moran’s *I* identifies statistically significant spatial units with a high number of cases that are surrounded by similar units (high–high, hereafter referred to as hotspots).^33^ Similarly, Getis-Ord Gi* identifies statistically significant hotspots where a specific location and its adjacent areas have values notably higher than the overall average value.^35^ To calculate both LISA methods, we applied the same parameters as for global Moran’s *I*. In addition, to decrease the false discovery rate (FDR) and control the expected rate of erroneously rejected hypotheses, we applied the FDR correction proposed by Benjamini and Hochberg^36^ to account for multiple and dependent tests in LISA.^37^ The spatial unit (neighborhood) was used as the geographical unit for this analysis according to the year of analysis. ArcGISPro 3.1.0 (ESRI, Redlands, CA) was used for SA and LISA analyses.

### Socioeconomic strata, land cover, and hotspot distribution.

We selected hotspots as those identified by both LISA methods and then, classified all of the spatial units into three classes: non-hotspots (NHHs), hotspots (HHs), and recurrent hotspots (RHHs; areas that were HHs for more than 2 years). We used a χ^2^ test (α = 0.05) to check the null hypothesis of even distribution of the classified spatial units into the socioeconomic strata previously defined. Finally, for comparing the land cover area proportion across the classified spatial units, we used the Kruskal–Wallis test (α = 0.05) for multiple comparisons with the Dunn test as post hoc tests,^38^ adjusting the *P*-values with the Benjamini and Hochberg^36^ method. We run the Kruskal–Wallis test in the Stats package v. 4.4.0 (R Foundation for Statistical Computing, Vienna, Austria), the Dunn test in the FSA package v. 0.9.5,^39^ and all of the processes in RStudio v. 2023.12.0 + 369 (http://www.posit.co/).^40^

## RESULTS

### Global SA.

We found that 50% of the dengue cases are concentrated in approximately 26% of the study area and a statistically significant positive SA throughout the entire study period (2010–2020; Moran’s *I* = 0.30, *z =* 8.67, *P* <0.001). Global Moran’s *I* ranged from 0.095 to 0.414 for individual years, showing statistical significance each year, except in 2011 and 2012 (Table 2). We found different spatial correlations of dengue cases across the years, with some of them having a high SA; in particular, SE and E years showed higher SA and higher strength (*z* value) than NE years.

**Table 2:** Number of hotspots and spatial autocorrelation of dengue cases in Medellin (Colombia) from 2010 to 2020 across severe epidemic, epidemic, and non-epidemic years

Year (epidemic level)	Cases Included in Analysis	Global Moran’s *I*	*Z* Value	Pseudo *P*-Value	Number of HotSpots (local Moran’s *I*)	Number of HotSpots (Getis-Ord Gi*)
2010 (SE)	13,945	0.316	8.521	0.001	28	31
2011 (NE)	595	0.029	0.715	0.474	9	11
2012 (NE)	552	−0.066	−1.382	0.167	3	4
2013 (E)	1,827	0.228	5.859	0.001	21	31
2014 (E)	2,676	0.290	7.731	0.001	20	29
2015 (E)	3,243	0.414	10.931	0.001	16	31
2016 (SE)	14,055	0.396	10.570	0.001	32	39
2017 (E)	1,655	0.138	3.604	0.001	10	20
2018 (NE)	917	0.095	2.354	0.018	13	20
2019 (NE)	909	0.131	3.175	0.002	9	20
2020 (NE)	440	0.156	3.427	0.001	6	6

E = epidemic; NE = non-epidemic; SE = Severe epidemic. See Table 1 for the epidemic-level classification.

### LISA analysis and hotspot identification.

**Figure 3.**
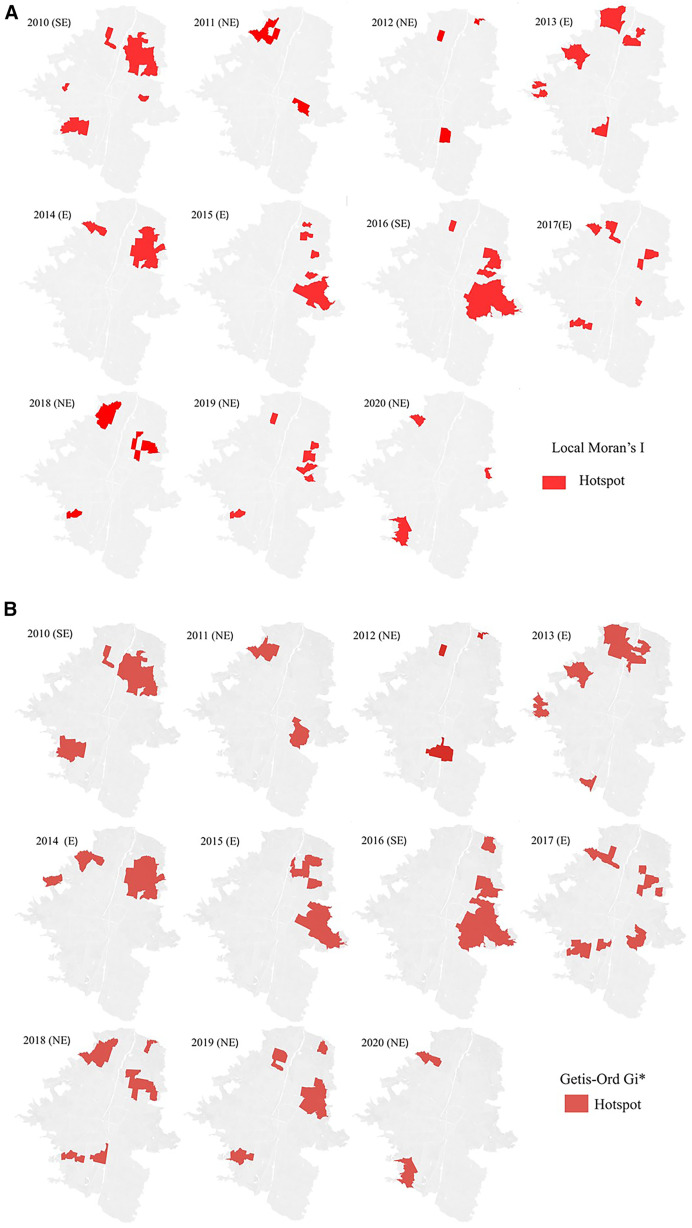
Dengue hotspots in Medellin (Colombia) from 2010 to 2020 based on (**A**) local Moran’s *I* and (**B**) Getis-Ord Gi*. E = epidemic; NE = non-epidemic; SE = severe epidemic.

Overall, the spatial patterns of HH found with both methods were similar (Figure 3), indicating that several HHs were in the northern and eastern areas of the city, although Getis identified more spatial units as HHs (Table 2). During SE years and E years, we observed an increase in the number of HHs, which were concentrated in specific areas of the city (Figure 3). In SE years, HHs were mostly found toward the northeast and central east of the city. Particularly, in 2010, two groups of them were identified; the largest group was in the northeastern area, whereas the other was in the southwestern area. We observed that during SE years, the two methods indicated the same focus area of HHs with a broader coverture using Getis (Figure 3). During E years, the pattern was “dispersed” (Figure 3); particularly, in 2013, hotspots were in the west, and in 2014 and 2015, they were in the east. Therefore, spatial pattern of hotspots changed over time. In 2013 and 2017, we observed small hotspot areas both in the north and in the south. However, in 2014 and 2015, with a noticeable absence of hotspots in the south, they were mainly found in the north and east of the city, affecting more spatial units compared with the other 2 NE years. Conversely, in NE years, hotspots were dispersed and concentrated in smaller areas throughout the city (Figure 3), most of them in the northeast and central east.

### Recurrence of dengue hotspots.

**Figure 4.**
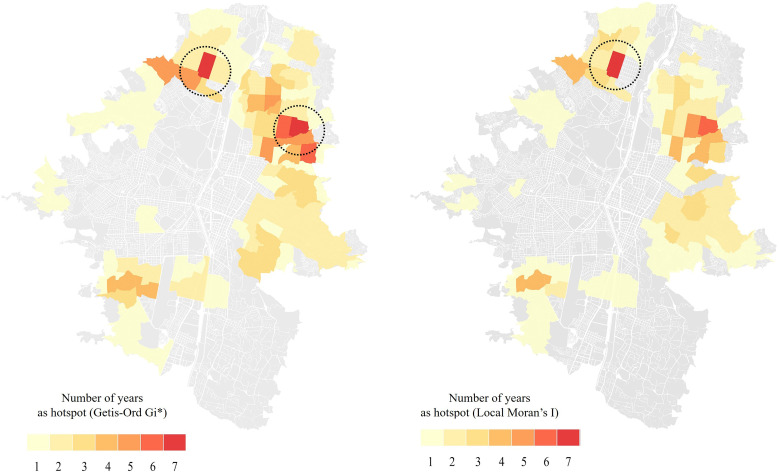
Recurrence of dengue hotspots in Medellin (Colombia) from 2010 to 2020 identified using Getis Ord Gi* (left panel) and local Moran’s *I* (right panel).

Throughout the study period, 168 HHs were identified using Moran’s *I* and 242 HHs were identified using Getis-Ord Gi* (Table 3; Figure 4). Of these, 91 HHs were common to both methods, with 47 classified as RHHs. The recurrence of HHs showed spatial persistence, with certain spatial units exhibiting as RHHs for up to 7 years (Figure 4). The northern and eastern areas showed a pronounced concentration of HHs and RHHs, where the spatial units were predominantly characterized by low socioeconomic strata.

**Table 3:** Statistical results comparing land cover categories and the distribution of spatial units classified as non-hotspots, dengue hotspots, and recurrent dengue hotspots in Medellin (Colombia) during 2010–2020 based on a Kruskal–Wallis test

Land Cover	Kruskal–Wallis (*H*)	df	*P*-Value
Agricultural	8.995	2	0.01
Water bodies	9.811	2	0.007
Artificial territories	21.213	2	<0.001
Forest and seminatural areas	18.886	2	<0.001

df = degree of freedom.

**Figure 5.**
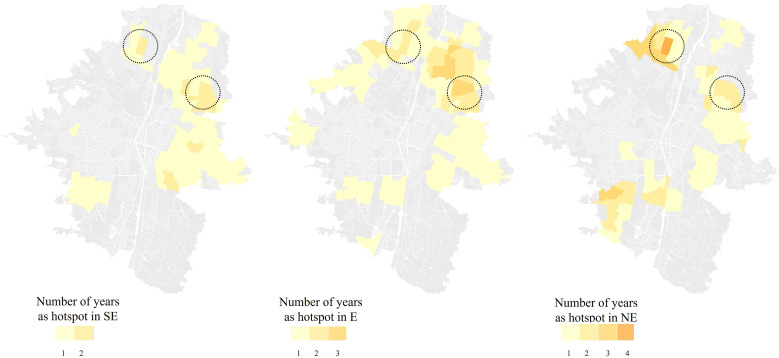
Recurrence of dengue hotspots in Medellin (Colombia) during 2 years of severe epidemic (SE), 4 years of epidemic (E), and 5 years of non-epidemic (NE) from 2010 to 2020. Colors indicate the number of years that each spatial unit was identified as a statistically significant hotspot.

The spatial patterns of HHs varied across SE, E, and NE years: 6 RHHs during the 2 SE years, 13 RHHs in the 5 NE years, and 18 RHHs in the 4 E years (Figure 5). Remarkably, some HHs recurred consistently regardless of epidemic level, appearing across SE, E, and NE years, particularly in the northern and eastern areas (Figure 5).

### Socioeconomic strata and land cover on hotspots distribution.

**Figure 6.**
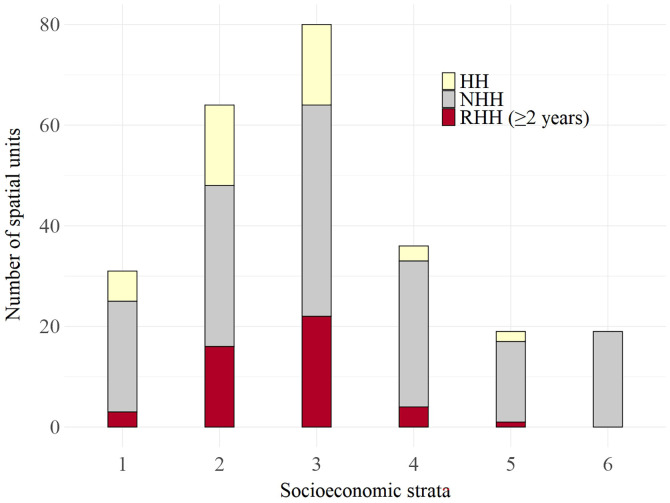
Dengue hotspots distribution across socioeconomic strata in Medellin (Colombia) from 2010 to 2020. Spatial units are classified as non-hotspots (NHHs), hotspots (HHs), and recurrent hotspots (RHHs).

We found a statistically significant difference in the distributions of HHs, NHHs, and RHHs across socioeconomic strata (χ^2^ = 18.99, degrees of freedom = 8, *P*-value = 0.01). Particularly, the distribution of RHHs also varied across the strata, being predominant in strata 2 and 3 (Figure 6). We noted a remarkable absence of HHs and RHHs in socioeconomic stratum 6 and rarely observed RHHs in stratum 5 (Figure 6).

**Figure 7.**
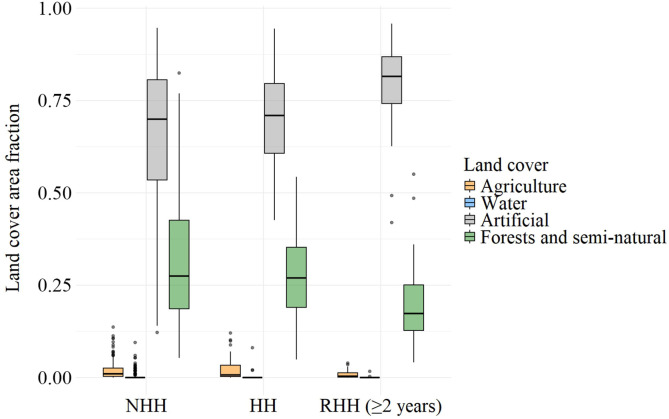
Land cover distribution in spatial units classified as non-hotspots (NHHs), hotspots (HHs), and recurrent hotspots (RHHs) in Medellin (Colombia) from 2010 to 2020. Box plots show the median (the horizontal line within each box), interquartile range (IQR; represented by the box), and whiskers extending to 1.5 times the IQR. Points beyond the whiskers represent outliers.

We found differences between RHHs and the other spatial units (NHHs and HHs) across agricultural, artificial, and forest land covers. Spatial units classified as RHHs had a higher proportion of artificial land cover and lower forest land cover compared with NHHs (Figure 7). For water bodies, only NHHs and RHHs showed significant differences. Notably, NHHs and HHs did not differ across any of the studied land covers (Table 4), suggesting a very similar distribution of land cover between these two spatial unit classes (Figure 7).

**Table 4:** Statistical results comparing land cover and the distribution of spatial units classified as non-hotspots, hotspots, and recurrent hotspots in Medellin (Colombia) from 2010 to 2020 based on a Kruskal–Wallis test followed by a Dunn test as post hoc tests

Land Cover	Comparison	*z*	*P*-Value (unadjusted)	*P*-Value (adjusted)
Agricultural	NHH–HH	−0.127	0.899	0.899
Agricultural	HH–RHH	2.218	0.026	0.040
Agricultural	NHH–RHH	2.951	0.003	0.009
Water bodies	NHH–HH	−2.080	0.038	0.056
Water bodies	HH–RHH	0.464	0.643	0.643
Water bodies	NHH–RHH	2.742	0.006	0.018
Artificial territories	NHH–HH	0.678	0.497	0.497
Artificial territories	HH–RHH	−3.061	0.002	0.003
Artificial territories	NHH–RHH	−4.593	<0.001	<0.001
Forest and seminatural areas	NHH–HH	−0.664	0.506	0.506
Forest and seminatural areas	HH–RHH	2.868	0.004	0.006
Forest and seminatural areas	NHH–RHH	4.333	<0.001	<0.001

HH = hotspot; NHH = non-hotspot; RHH = recurrent hotspot.

## DISCUSSION

We studied the spatiotemporal dynamic and recurrence of dengue hotspots across SE, E, and NE years from 2010 to 2020 in Medellin. We found statistically significant hotspots, some of them recurring for up to 7 years and exhibiting different spatial patterns according to the SE, E, and NE years. Our results reveal significant insights into the spatial dynamics of dengue, which might be important for public health interventions and control strategies in the city based on evidence.

### Global and local SA.

Our finding of years with high SA during E and SE years agrees with other research in dengue and malaria.^41^^,^^42^ This strong SA may result from dengue cases clustering in specific geographic areas during epidemics, influenced by factors such as a high population density, favorable environmental conditions for mosquito breeding, human movement patterns, and socioeconomic conditions.^18^^,^^26^^,^^43^ These clusters heightened the SA as neighboring areas are more likely to exhibit similar disease patterns, contributing to the occurrence of HHs.

Although there were similarities in the patterns of HHs using both LISA methods, differences emerged in their number, with Getis-Ord Gi* indicating a higher number of HHs. The similarities in spatial patterns between methods are consistent as well as the variations in the numbers of HHs for malaria,^44^ coronavirus disease 2019,^45^ and dengue.^46^ Although local Moran’s *I* and Getis-Ord Gi* are similar in terms of the types of questions that they address, they have key differences that may explain why we obtain different results. The main difference lies in how they calculate the local mean as indicated in the Materials and Methods section.

### Recurrence of dengue hotspots.

The variability of HHs over time and across the city is consistent with previous research, indicating that transmission of dengue is highly heterogeneous in terms of space and time.^11^ Our study reinforces previous evidence that significant proportions of dengue cases are concentrated in small areas within urban areas and that these areas are recurrent hotspots throughout the years.^10^^,^^26^^–^^28^^,^^47^ Particularly, 50% of the dengue cases in Medellin clustered in 26% of the study area, concentrated in the north and eastern areas of the city. This pattern has also been found in other cities, although the proportion of dengue cases within hotspots varies significantly among them.^27^^,^^47^ For instance, in Iguala (Mexico), around 50% of cases were concentrated within 23% of the city,^26^ whereas in Tartagal (Argentina), around 50% of cases clustered within 35.9% of the urban area.^27^ This highlights that targeted interventions in a portion of the dengue-affected area could significantly reduce the number of cases as noted in refs. 8 and 11. However, the challenge lies in finding the right locations for these interventions, which our study addresses by identifying statistically significant hotspots.

The variation in the spatial distribution of hotspots across SE, E, and NE years was expected. This finding is consistent with studies conducted in other cities, where the spatial patterns of dengue varied depending on the epidemic level.^26^^,^^48^ In an epidemic, the higher number of infected individuals typically leads to more pronounced and concentrated hotspots. This pattern is common in diseases, where outbreaks result in localized clusters of cases as transmission accelerates in densely populated or vulnerable areas.^26^

The temporal recurrence of hotspots has also been identified in other cities.^10^^,^^26^^–^^28^ Targeted interventions and proactive measures could be implemented in these areas as a strategy to efficiently use resources and decrease the burden of dengue, especially in hyperendemic cities.^11^ The remarkable recurrence of some HHs in this study raises important questions. Were these areas targeted during epidemic years? Is there any specific preventive intervention that could help reduce the recurrence of dengue in these neighborhoods? One possible answer is that these regions might have been identified too late, possible because of challenges faced by the administration of public health systems. Targeting recurrent hotspots with preventive and control measures could offer valuable insights into the effectiveness of this strategy to reduce the number of dengue cases. Therefore, evaluating the epidemiological impact of focusing on these RHHs and comparing it with the effectiveness of reactive approaches are necessary.

### Socioeconomic strata, land cover, and hotspots.

We found that socioeconomic strata are related to the presence of HHs and RHHs, similar to cities in Mexico,^26^ and also have been related to dengue outbreaks.^18^^,^^49^^–^^51^ People in low socioeconomic strata may face greater risks than those living in higher strata because of a combination of factors, such as limited access to resources, inadequate infrastructure, and higher population densities. Poor waste management, inadequate water storage practices, and a lack of effective mosquito control measures create a more favorable environment for the breeding and survival of vectors.^52^ These factors highlight the necessity for targeted public health interventions that address the specific vulnerabilities of these communities.

The lack of statistical difference between NHHs and HHs was an unexpected result, suggesting that HHs may form across any combination of land cover types observed in our study. In contrast, RHHs showed a significant difference compared with both HHs and NHHs. Similar to findings in other cities with high dengue incidence,^53^ we identified RHHs associated with low forest and seminatural land cover and higher artificial land cover. In low socioeconomic strata, areas with these characteristics—larger built-up areas with few green spaces—are common, creating conditions that enhance contact between infected *Ae. aegypti* mosquitoes and humans,^53^ which may lead to increased dengue cases.

Given that *Ae. aegypti* prefers to stay in built-up areas and breed in artificial containers,^52^ this could explain the association between RHHs and high artificial land cover. However, *Ae. albopictus*, which prefers to breed in natural habitats (such as tree holes, bamboos, stumps, and bromeliads),^54^ also coexists in the area^55^ and has been found infected with dengue and Zika viruses.^56^^,^^57^ All of these factors lead us to question whether the role of *Ae. albopictus* in transmitting dengue is minimal in RHHs. Unfortunately, the epidemiological significance of *Ae. albopictus* in dengue transmission remains unclear. To address this, it is essential to explore how these vectors interact with different land cover and contribute to dengue transmission in the area.

It is also important to consider that land cover and socioeconomic strata are related to the study area. Although we did not assess their relationship, it is possible to see how the higher strata have the largest area with forest and seminatural forest cover over the city (Figure 1), a pattern previously identified in the city.^58^ Also, vegetation richness and abundance^59^ are known to vary across socioeconomic gradients, which further contributes to the local breeding sites of the dengue vectors as discussed in the previous paragraph. Understanding the interaction between socioeconomic and land cover variables and their influence on the presence and recurrence of RHHs would be valuable for designing targeted control strategies.

### Management implications.

Hotspots are a consistent feature of dengue transmission at all endemicities,^26^^,^^28^ and their identification is the first step for constructing evidence of the efficacy of the risk stratification approach to lower the dengue burden. Targeting interventions in hotspots could potentially achieve a substantial decrease in dengue transmission, surpassing the effectiveness of random interventions as previous mathematical models have suggested,^60^ although there is a lack of research indicating the effectiveness of such interventions. For considering it as a direct recommendation for decision-makers, direct evidence is needed, even though it would be difficult to change the settings for the strategies that governments need to follow to control dengue. Targeting recurrent hotspots could be a measure paying off because these areas may seed transmission in the surrounding area as studies in other mosquito-borne diseases, such as malaria, have suggested.^61^^,^^62^

Our results support the need to optimize control strategies for dengue transmission by implementing novel approaches, and spatially targeted interventions in specific hotspot areas could be crucial. In Medellin, novel strategies to control dengue have been implemented in the last few years. For instance, entomovirological surveillance has become a routine activity conducted by the municipality’s Secretary of Health to detect early arbovirus circulation.^56^^,^^63^ Also, from 2017 to 2022, *Wolbachia*-infected mosquitoes were released as a strategy to reduce the dengue incidence. Preliminary results showed promising trends in *Wolbachia* establishment and a reduction in dengue incidence.^64^^,^^65^ However, after the final periods of release, low *Wolbachia* prevalence was found in mosquito populations.^66^

The implementation of these novel dengue control strategies could also be optimized by prioritizing their initial rollout in hotspot areas, where the potential is greater for short-term benefits, and the prioritization of hotspots for initial rollout should be considered over the implementation in lower-risk zones.

### Limitations.

We acknowledge certain limitations in our research. Our focus on symptomatic dengue cases may not fully capture the impact of hotspots as asymptomatic cases also contribute to disease transmission. Additionally, not all cases were confirmed through laboratory testing, which may lead to misdiagnoses because of similarities with other mosquito-borne diseases, like Zika and chikungunya; however, the available information does not allow us to perform such analysis.

We found differences in dengue case numbers between our study and others.^50^^,^^67^ They stem from variations in how the cases are reported and managed within the surveillance system. When a suspected dengue case is identified, it goes through multiple levels—from local units (Unidad Primaria Generadora del Dato, in Spanish) to international agencies, like Pan American Health Organization and WHO. During this process, cases may be confirmed through laboratory tests or removed because of duplicates, affecting the overall count.^68^ Considering the complexity of compiling and maintaining such datasets, continuous improvements are necessary to enhance the reliability of decision-making.

Medellin is situated at the epicenter of a metropolitan region surrounded by neighboring municipalities with high incidences of dengue.^69^ We identified hotspots in the peripheral areas to the north and east of Medellin; however, we did not study the interplay with the surrounding areas of the metropolitan area. Additionally, by using only residential addresses, we are unable to identify out-of-home locations where infections might have occurred.^70^ Human mobility plays a crucial role in the dynamics of dengue transmission, with frequent short-range movement within neighborhoods significantly contributing to the local spread.^70^ Future studies of human mobility in Medellin will help clarify the role of hotspots as foci of infection for both locals and visitors.^71^ Also, previous studies in Medellin showed that the spatial pattern of dengue was driven by public transit development.^43^ Broadening the study area and compiling additional information would provide a more in-depth understanding of the dynamics influencing dengue transmission in a metropolitan area.

Our study is the first approach using dengue cases to identify hotspots; future multicomponent models to find high-risk areas could better inform public health interventions and mitigate the impact of dengue.^50^^,^^72^

## CONCLUSION

This research highlights the complexity of dengue transmission dynamics in a hyperendemic city. The recurrence of dengue hotspots is a persistent characteristic of endemic cities. These hotspots are associated with socioeconomic strata and land cover variables, with notable variations across both epidemic and non-epidemic years. This suggests a complex interplay between these factors, which needs more robust analysis and careful consideration in public health planning. Although we do not have empirical evidence directly showing that targeting RHHs would reduce the burden of dengue, our results present an opportunity to evaluate whether targeted control measures in hotspot areas are more effective than broad, wide-ranging interventions in managing dengue.

## Data Availability

Socioeconomic and land cover data were obtained from open data sources available through the Medellin Health Department Portal: https://www.medellin.gov.co/giscatalogacion/srv/spa/catalog.search#/home and https://www.medellin.gov.co/irj/portal/medellin?NavigationTarget=contenido/6989-Geomedellin-el-portal-de-datos-geograficos-del-Municipio-de-Medellin. Dengue case data are proprietary and available upon request from the Medellín City Secretariat of Health subject to their discretion.
